# Quality Assessment of Investigational Medicinal Products in COVID-19 Clinical Trials: One Year of Activity at the Clinical Trials Office

**DOI:** 10.3390/ph14121321

**Published:** 2021-12-17

**Authors:** Diego Alejandro Dri, Giulia Praticò, Elisa Gaucci, Carlotta Marianecci, Donatella Gramaglia

**Affiliations:** 1 Clinical Trials Office, Italian Medicines Agency (AIFA), Via del Tritone 181, 00187 Rome, Italy; g.pratico@aifa.gov.it (G.P.); e.gaucci@aifa.gov.it (E.G.); d.gramaglia@aifa.gov.it (D.G.); 2 Department of Drug Chemistry and Technologies (DCTF), Sapienza, University of Rome, Piazzale Aldo Moro 5, 00185 Rome, Italy

**Keywords:** clinical trials, COVID-19, investigational medicinal products, quality, regulatory

## Abstract

One year after the spread of the pandemic, we analyzed the assessment results of the quality documentation submitted to the Clinical Trials Office of the Italian Medicines Agency as part of the request for authorization of clinical trials with a COVID-19 indication. In this article, we report the classification of the documentation type, an overview of the assessment results, and the related issues focusing on the most frequently detected ones. Relevant data regarding the Investigational Medicinal Products (IMPs) tested in COVID-19 clinical trials and their quality profiles are provided in the perspective of increasing transparency and availability of information. Some criticalities that have been exacerbated by the management of clinical trials during the emergency period are highlighted. Results confirm that IMPs tested in authorized COVID-19 clinical trials are developed in agreement with the same legal requirements for quality, safety, and efficacy as for any other medicinal product in the European Union (EU). The same strong regulatory framework applies, and there is no lowering in the safety profile due to the pandemic; authorized IMPs meet the highest standards of quality. The regulatory network should capitalize on lessons learned from the emergency setting. Some take-home messages are provided that could support the regulatory framework to expand its boundaries by innovating and evolving even though remaining strong and effective.

## 1. Introduction

The first evidence of a COVID-19 infection in Italy was registered by the Italian National Health System on 21 February 2020. Soon after this case was detected in Lombardy, the pandemic spread throughout the country. On 6 March 2020, the Clinical Trials Office (CTO) at the Italian Medicines Agency (AIFA) received the first applications of COVID-19 clinical trials (CTs), submitted through the national system Osservatorio Nazionale delle Sperimentazioni Cliniche (OsSC) [1].

On 17 March 2020, the Italian Government issued a decree-law that, limited to the emergency period, imposed all CTs from Phase I to IV, observational studies, and compassionate-use therapeutic programs to be preliminarily evaluated by the technical scientific commission of AIFA [2]. The number of requests for authorization of CTs with a COVID-19 indication rapidly increased, and the CTO, despite the emergency, continued to guarantee the service supporting the protection of public health. CT applications were timely assessed from an administrative, regulatory, non-clinical, clinical, statistical, and quality perspective.

**Figure 1:**
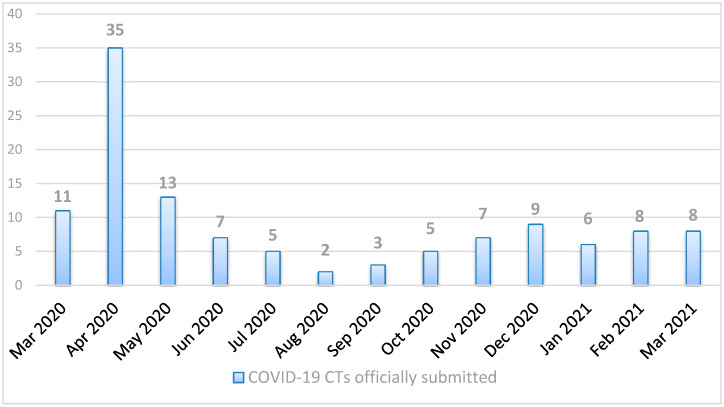
COVID-19 CTs officially submitted with a unique EudraCT number to the CTO on a monthly basis from March 2020 to March 2021.

In Figure 1, we report the number of COVID-19 CT submissions officially received with a EudraCT number [3] at the CTO from March 2020 to March 2021.

In April 2020, immediately after the spread of the pandemic, there was an increase in the number of submissions, followed by a progressive decrease during the summer period when the epidemiological situation in the country had improved. Unfortunately, in October 2020, the number of daily infections in the country increased again, and the number of submissions reflected the trend.

Before a medicinal product is granted marketing authorization in the European Union (EU), CTs need to be performed to define or confirm the safety and efficacy profile of Investigational Medicinal Products (IMPs). CTs must comply with specific guidelines in the EU [4], and the assessors of the national competent authorities (NCAs) are responsible for the assessment of the benefit-risk ratio of the clinical study protocols and IMPs. Accurate physicochemical characterization and the control of critical quality attributes (CQAs) play a crucial role in establishing that the safety profile is ensured.

In this article, for the first time, we retrieve, analyze, and discuss the quality documentation and data provided by sponsors in the clinical trial applications (CTA) with a COVID-19 indication submitted to the CTO during the first year of the pandemic. According to assessors’ experience, potential differences may apply depending on the nature of the sponsor; therefore, we also wanted to investigate if quality issues might be more common with commercial than with non-commercial trials. The pool of data includes the very first COVID-19 CTs received in March 2020 and contains all those received subsequently up to March 2021. Due to the potential commercially confidential nature of most of the information managed, full data cannot be disclosed, and results are provided as aggregated data. We also identify some lessons learned and take-home messages for further regulatory reflections.

## 2. Results

**Figure 2:**
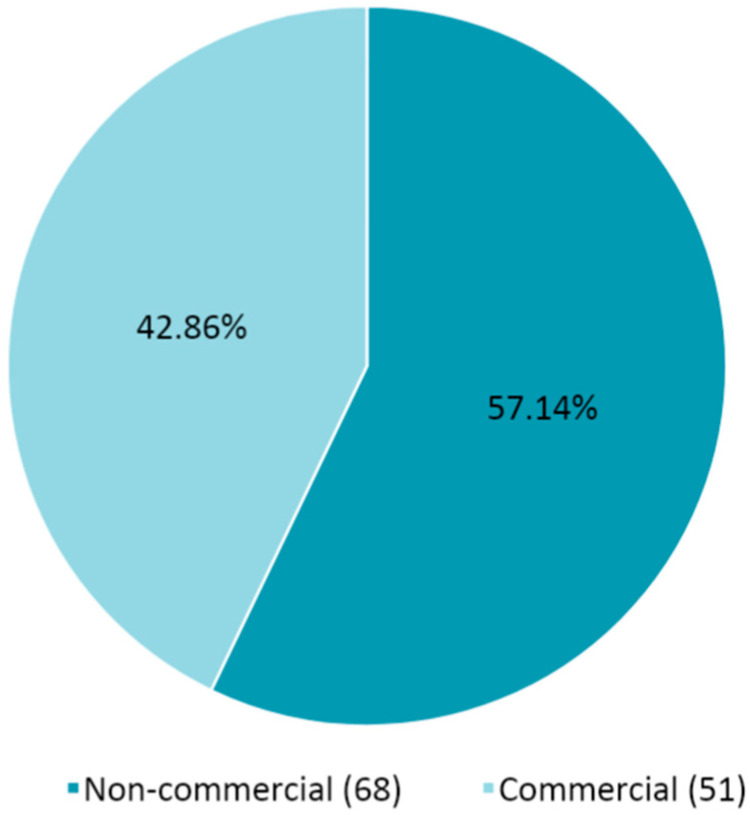
Percentage (number) of commercial and non-commercial COVID-19 CTs assessed from March 2020 to March 2021.

According to the information declared in the submission package of the 119 EudraCTs submitted in the mentioned timeframe, the percentage of commercial or non-commercial CTs is provided in Figure 2.

The number of non-commercial CTs is about 14% higher than the commercial ones.

The number of COVID-19 CTs applications assessed from March 2020 to March 2021 at the CTO, differentiated per study type and quality assessment outcome, is summarized in Table 1.

Overall, quality issues were raised for 85 CTs (71.43%). In absolute terms, the number of CTs experiencing quality issues is comparable across study types. Quality issues were recorded for 44 non-commercial CTs and for 41 commercial CTs. However, in terms of percentages for a given study type, 80.39% of commercial studies were impacted by quality issues versus 64.71% of non-commercial studies. This result could be explained if read in conjunction with the analysis of the type of documentation presented, mainly summary of product characteristics (SmPCs) for non-commercial studies and full investigational medicinal product dossier (IMPDs) for commercial ones.

The number of non-commercial studies that did not experience any quality issue (24 CTs) is more than twice the number of commercial ones (10 CTs), while in terms of percentage per study type, 35.29% of non-commercial studies did not experience any quality issues versus only the 19.61% of commercial studies. Considering that non-commercial studies usually investigate authorized IMPs with a consolidated quality and safety profile, a higher percentage of non-commercial CTs without quality issues was indeed expected.

### 2.1. Quality Documentation

**Figure 3:**
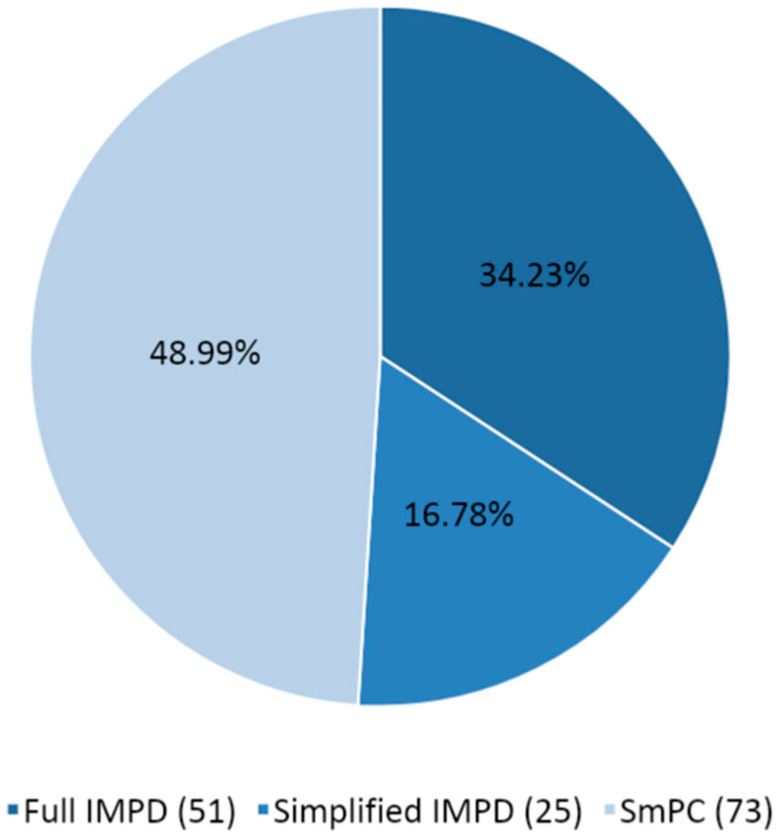
Percentages (number) of the different types of quality documentation for COVID-19 CTs assessed from March 2020 to March 2021.

Figure 3 shows the type of quality documentation submitted as part of the CTAs assessed from March 2020 to March 2021, including the number of instances and the percentages.

It should be noted that when the same IMP is tested and assessed in the context of different CTs, the IMP is counted multiple times for the purpose of identifying the type of quality documentation submitted, and in the same CT, the IMP could be counted multiple times if multiple pharmaceutical forms or strengths were tested. For a given CT, it is also possible to retrieve more than one IMP tested in the same study. The number of IMPs tested and related documentation is therefore higher than the number of CTs submitted. SmPCs (48.99%) are more frequently submitted, followed by full IMPDs (34.23%) and simplified IMPDs (S-IMPDs) (16.78%). SmPCs and S-IMPDs together count two-thirds of the overall submitted documentation (65.77%). This reflects the fact that most of the IMPs already had a marketing authorization and that they were therefore investigated with a repurposing scope. This is in line with the expected behavior during a health emergency and the urgent need to identify an effective treatment, capitalizing on already marketed products.

**Figure 4:**
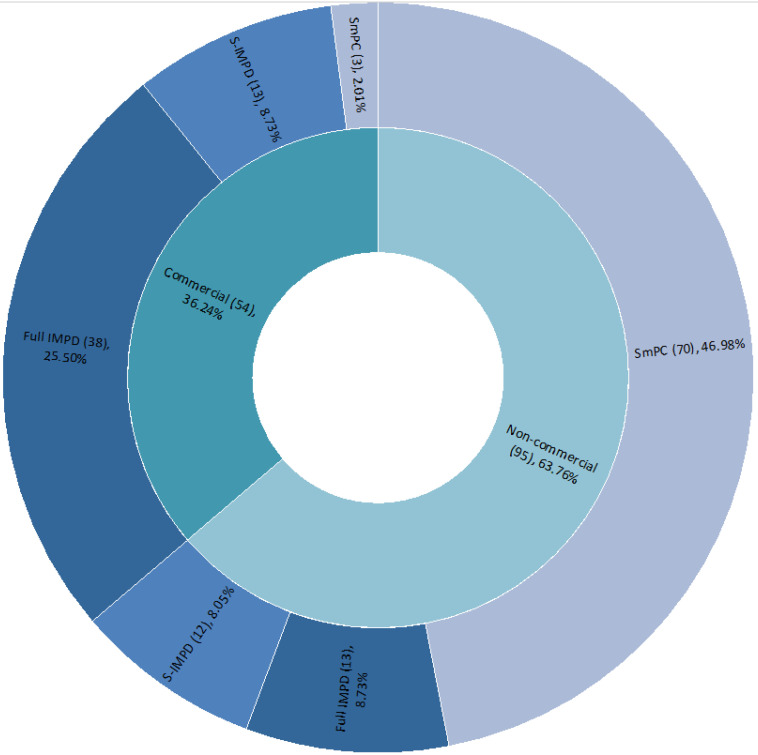
Percentage (number) of quality documentation types for commercial and non-commercial COVID-19 CTs assessed from March 2020 to March 2021.

Further analysis shows that, depending on the commercial or non-commercial nature of the study, a specific document-type stratification can be observed (Figure 4).

Only considering non-commercial CTs, the quality documentation consists mainly of SmPCs (73.68%), while only considering commercial CTs, most of the quality documentation is represented by full IMPDs (70.37%). This is in line with the consolidated safety and quality profile of the IMPs expected to be tested in CTs, especially by non-commercial sponsors, considering that usually, they are not the marketing authorization holders of the medicinal products.

There is no difference in the percentage of S-IMPDs across commercial or non-commercial studies.

### 2.2. Quality Issues

Quality issues raised on IMPs involved in COVID-19 CTs from March 2020 to March 2021, and their classification according to the currently applicable guidelines [5,6], are detailed in Table A1, compared across commercial and non-commercial study types.

A total of 665 issues have been globally raised, including objections, requests for clarification, requests for updated documentation or data, requests for additional information, conditions, and recommendations regarding quality. Issues were raised in 85 out of 119 CTs (71.43%), with an average of 7.82 issues per CT. It is important to note that issues were retrieved for almost all categories, except for drug substance nomenclature and drug product characterization of impurities, where a cross-reference to data in the drug substance section of the IMPD is usually adopted.

A total of 525 (78.95%) issues were retrieved for commercial CTs and only 140 (21.05%) for non-commercial ones, with an average of 12.80 issues per commercial CT and only 3.18 issues per non-commercial CT. This is potentially related to the fact that non-commercial CTs IMPs with a consolidated benefit-risk profile are mainly tested. However, in four categories, the number of raised issues for non-commercial CTs is higher than that for commercial ones: CTA forms compliance, quality documentation compliance, control of excipients, and labeling. Issues regarding CTA form and quality documentation compliance might denote not fully consolidated administrative and regulatory expertise.

**Figure 5:**
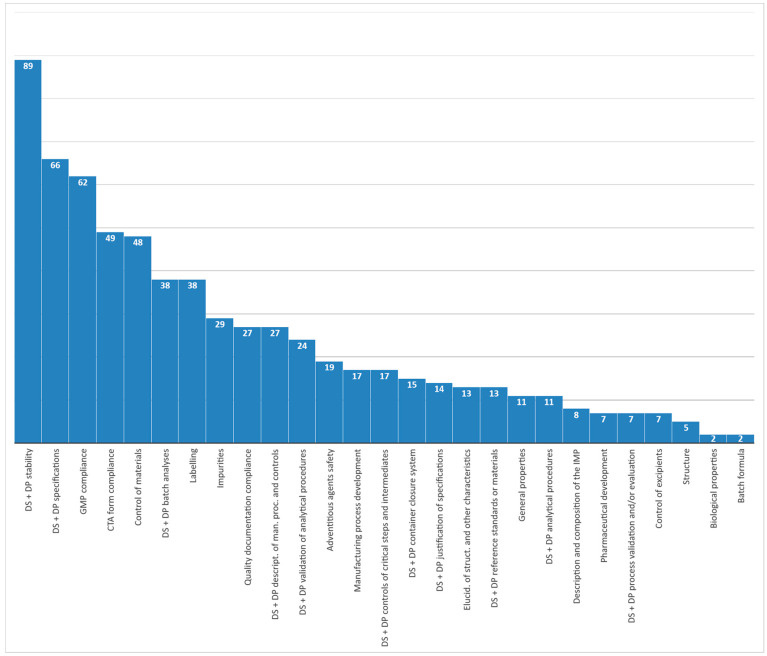
Number of quality issues combining drug substance (DS) and drug product (DP) classification label in COVID-19 CTs assessed from March 2020 to March 2021.

The highest number of criticalities were found in specific areas, such as Good Manufacturing Practice (GMP) compliance, CTA form compliance, control of materials, labeling, impurities, and quality documentation compliance. However, if we cumulate issues for the same classification label, combining the drug substance and the drug product sections of the IMPDs, a description of the manufacturing process and process controls and batch analyses gains more weight. However, the largest number of issues is clearly related to stability and specifications. Figure 5 provides additional details on those categories where the number of quality issues was higher (≥27).

#### 2.2.1. Stability

Stability issues impact to the largest extent in terms of absolute numbers considering both the drug substance and drug product sections of the IMPDs. The most common findings are related to the lack of data or insufficient stability data provided by the sponsors to support a proposed retest date or shelf life. However, upon request for additional supporting data, sponsors were usually able to provide updated data, and the issue could be considered closed. Nevertheless, in some instances, critical trends were noted in data deriving from stability studies, and therefore limitations to proposed retest or shelf-life were implemented.

#### 2.2.2. Specifications

The control of drug substances and drug products requires the setting of proper specifications and relevant tests, including the acceptance criteria, that is critical information that should be specified for the batches intended to be used in the CT. Upper limits are often not fully justified, and safety considerations are not taken into account when setting the limits for impurities, with a special focus on potential mutagenic ones. Additional criticalities are found when addressing the microbiological quality of drug substances used in aseptically manufactured products.

#### 2.2.3. GMP Compliance

Most GMP issues relate to the missing evidence of manufacturing and importation authorization or GMP certificates of manufacturers involved in the CTs. Information not properly compiled or inconsistently reported in qualified person (QP) declarations are also a recurrent issue. In addition, due to the need to use IMPs not manufactured in the EU during the emergency setting, in some cases, the pharmacies of the CTs sites have taken the responsibility to import the IMP into the EU, as allowed by national law [7] for non-commercial CTs.

#### 2.2.4. Control of Materials

This classification label includes a heterogeneous list of issues. Examples are: missing identification of the starting materials, or, when they are identified, critical attributes or specifications are not reported or accurately described, such as in the case of acceptance limits for appearance or assay; specifications proposed for the starting material not in agreement with the proposed CT phase; intermediates and/or impurities not fully controlled or with missing structural formula; grade of reagents and solvents used in the manufacture of the drug substance not provided and, in case recycled solvents are used (e.g., N,N-Dimethylformamide (DMF)), as this may pose a risk for nitrosamine formation, additional information and clarification are not provided; when compendial materials are used and tested according to in-house specifications, these are not provided; when impurities in drug substance derives from the starting materials, the control of impurities in starting materials is not always considered; missing Transmissible Spongiform Encephalopathy (TSE) and Bovine Spongiform Encephalopathy (BSE) certification for materials of animal origin; benzene not properly controlled in the solvents or in the manufacturing process.

#### 2.2.5. CTA Form Compliance

Properly compiling the CTA form has always been a criticality, but the number of issues increased during the evaluation of COVID-19 CTs. Most of the detected issues are related to section D (information on each IMP), with special reference to dosages, dosage strength information, and sites where the QP certifies the batch release. A higher incidence was observed in the number of issues detected for non-commercial CTs. Most of the issues were solved by asking and supporting the sponsors to comply with the dedicated guideline already implemented by the CTO [8].

#### 2.2.6. Labeling

According to the CT Directive in EU [9], the labeling was expected to be at least in the official language(s) of the Member State on the outer packaging of the IMPs or, where there was no outer packaging, on the immediate packaging. During the assessment of COVID-19 CTs, the missing availability of correct labeling was a recurring criticality that would have delayed the authorization process and that therefore triggered the need to put in place temporarily urgent supportive actions coded in detailed specific guidelines by European Medicines Agency (EMA), AIFA [10,11], and most NCAs in EU. Procedures and logistics to label new IMPs for investigational purposes might, in fact, be extremely time-consuming, delaying the shipment of drug supplies. Issues in labeling during the pandemic were therefore expected to be mainly related to the unavailability of translations in the local language. However, looking at the data, the majority of issues were encountered for non-commercial studies, and these were related to non-compliance with Annex 13 to the EU Guidelines on GMP [12].

#### 2.2.7. Batch Analyses

Issues were mainly related to missing data for representative batches or to the presentation of old data (e.g., dated 2003, 2010, 2011), and therefore certificates of analysis of the most recent representative batches had to be requested. Information on which drug substance lots have been used for the manufacture of drug product batches intended to be used in the CT, or representative batches from all manufacturing sites or representative of the actual manufacturing process, was often missing and had to be requested.

#### 2.2.8. Impurities

Almost all issues were raised for CTs submitting full IMPDs. It is acknowledged that the necessary level of detail may be dependent on the phase of the CTs, and this is in line with the expected progress during the development phase of a new IMP and the continuous know-how-acquiring process in the synthetic route of manufacturing. However, the impurity profile (e.g., impurities, elemental impurities, solvents) is not always fully controlled and justified according to International Council for Harmonization (ICH) quality guidelines (e.g., Q3A, Q3D, Q3C) and often additional discussion, such as on potential mutagenic impurities according to ICH M7, needs to be provided (structure, origin, limit justification). The absence of routine control for solvents/catalysts used in the manufacturing process is also often not fully justified, or supportive data from batch analyses demonstrating effective purging of impurities are not provided. Further attention was posed on two additional and frequent topics: the control strategy for benzene and the lack of information about the synthesis of the starting materials, with the relative control of potential carry-over in the drug substance.

#### 2.2.9. Quality Documentation Compliance

Almost all issues in this category were connected to missing SmPCs or to the submission of unrepresentative ones for the IMPs to be used in the CT, such as language issues or submission of SmPCs of a medicinal product not authorized in the EU or in an ICH country. In some cases, issues were raised from the fact that, due to shortage of marketed products, the medicinal products used were equivalent products from non-EU or non-ICH markets, and for those, a full IMPD and full GMP compliance documentation were required. Occasionally, the IMPD format and/or overall content was also found to not be compliant.

#### 2.2.10. Description of the Manufacturing Process and Process Controls

Depending on the manufacturing process adopted, the number and type of findings may vary. However, the following recurrent issues are observed, in particular for aseptic and sterilizing processes: the state of validation of the aseptic process and lyophilization are not described; the validation of sterilizing processes, which should be of the same standard as for product authorized for marketing, is not discussed; justification for the filter integrity testing conducted only after use is not provided; the maximum time between the start of bulk solution preparation and sterile filtration is not defined and minimized; no information on the holding time between the end of filtration and filling in the final container, or on bioburden control before the second filtration.

## 3. Discussion

Quality issues were raised for 85 CTs (71.43%) out of 119 COVID-19 CTs received at the AIFA’s CTO from March 2020 to March 2021, with an overall average of 7.82 issues per CT. The majority (78.95%) of them was raised in the context of the assessment of commercial studies, where the quality documentation type was mainly full IMPDs (70.37%), while significantly fewer quality issues (21.05%) were detected for non-commercial studies, where the quality documentation type was mainly SmPCs (73.68%). This is in line with the expected consolidated safety and quality profile of the IMPs with a marketing authorization, investigated with a repurposing scope mainly in non-commercial CTs.

Almost two-thirds of the overall quality documentation (65.77%) is composed of SmPCs and S-IMPDs, reflecting a well-established safety and quality profile for most of the IMPs tested.

Quality issues impacted almost all sections of the IMPD, highlighting that the level of accuracy in the preparation of the quality documentation by sponsors is not always aligned to quality standards; the level of compliance should be increased across all sections.

Considering both the drug substance and the drug product sections of the IMPD, the highest number of quality issues was retrieved in the areas of stability data and specifications, which would require major attention from sponsors. GMP compliance, CTA form compliance, and control of materials are the three other categories collecting most issues. However, issues regarding batch analyses, labeling, impurities, quality documentation compliance, and description of the manufacturing process and process controls impacted to a great extent and are additional sectors that should also be closely monitored. Non-commercial sponsors had more difficulty in providing the quality documentation to support CT applications for the following issue categories: CTA form compliance, GMP compliance, quality documentation compliance, and labeling.

It is worth remembering the call from EMA’s Human Medicines Committee (CHMP) to pool research resources into large, multi-center, multi-arm clinical trials during the pandemic [13], to capitalize on available resources, and particularly to limit small studies or compassionate-use programs in EU that would not be able to provide the required level of evidence and that would not be in the best interest of patients. Likewise, CTS Guidelines on the submission of COVID-19 CTs [14] indicated that most of the CTs initially submitted came from academic sponsors, or in any case, not commercial.

It is crucial to capitalize on feedback received during the management of CTs in an emergency setting and to envisage dedicated support to micro-, small-, and medium-sized enterprises (SMEs) and non-commercial (academic) sponsors, particularly in view of the application of EU Regulation 536/2014 [15] so that the contribution provided to patient healthcare is not penalized.

Labeling issues experienced with the implementation of CTs during the pandemic, especially by non-commercial sponsors, clearly show how a more flexible approach from the regulatory network may be considered to streamline some processes, even though patients’ safety should always be prioritized.

The need to import IMPs that are not manufactured in the EU during an emergency situation also stressed the GMP regulatory boundaries and compliance.

Another bursting topic for further regulatory discussion is the implementation of decentralized procedures in a CT setting that definitely supported the implementation of CTs in the EU during the pandemic. Decentralized CTs need to be framed into a solid and consistent regulatory framework to support further innovative approaches to the conduction of CTs even after the end of the emergency. The message was received, some NCAs already reacted [16], and the clinical trials’ facilitation and coordination group (CTFG) is indeed already working on a recommendations document to capitalize on the lived experience.

It must be acknowledged that the EU network immediately reacted to critical issues during the management of CTs in the COVID-19 emergency setting. Take-home messages regarding the management of CTs in an emergency should be captured by the regulatory network to capitalize on the current experience and code lessons learned in a best-practice document or in a dedicated guideline for the conduction of CTs.

It is also important to understand that a more proactive approach, a prompt reaction to the challenges that the speed and increasing complexity of technological innovations are bringing along, and that are already impacting CTs (e.g., nanomedicines, decentralized trials, big data, artificial intelligence, and machine learning), should be part of a new and renewed regulatory mindset. The CTO is promptly reacting to innovation already impacting CTs today. As an example, a dedicated guide for the submission of a request for authorization of a CT involving the use of artificial intelligence (AI) or machine learning (ML) systems was recently published [17]; however, a global EU approach would be highly desirable. Increased involvement of stakeholders and the technological, academic, and scientific community, capitalizing on their know-how and expertise, should be envisaged to support the evolution and at the same time the reinforcement of the regulatory system, which in this way could be much more suitable to face future challenges.

## 4. Materials and Methods

The analysis was performed taking into consideration the interventional CTs with a COVID-19 indication officially submitted to the CTO in AIFA in one-year timeframe, from March 2020 to March 2021. Upon a preliminary review by the technical scientific commission of AIFA, and depending on its positive, suspensive or negative opinion, the assessment of the protocol and of the documentation included in the submission package may proceed according to the currently applicable regulatory framework [9] and the application is further evaluated by both the NCA and the Ethics Committee. The CT is considered authorized and can start the enrollment only after both the Ethics Committee provides a positive opinion and the NCA provides the authorization. A list with updated information on ongoing COVID-19 CTs and related documents is publicly available and can be consulted through the AIFA institutional website [18]. Data were obtained by consultation of AIFA’s CTO internal database, which were subsequently matched with the documentation uploaded either in OsSC or submitted to CTO by other means allowed due to emergency situation (e.g., paper submission or certified e-mail) [19]. Only CTs validated by CTO between March 2020 and March 2021, and officially registered with a unique EudraCT number, were considered. Information concerning IMP-related documentation was extracted by the CTA forms (Appendix A) of the selected CT applications. In our analysis, all CTs whose quality part was assessed were considered, regardless of whether the study has been ultimately authorized by the NCA or not, an ethical opinion was issued or not, or if the study was withdrawn. Based on the initial quality assessment performed at the NCA, if deemed necessary, additional requests for information or data are sent to the sponsors or grounds for non-acceptance issued, and based on the received responses, a final conclusion on the quality part of the dossier is adopted. The quality conclusion is then integrated as part of the overall assessment, including other sections of the dossier, such as regulatory, clinical, statistical, allowing a final decision to be issued by the NCA. The overall process should be concluded within 60 days, clock-stop excluded. During the pandemic, every effort was made to prioritize COVID-19 trials and shorten the above-mentioned timelines as much as possible.

The total number of COVID-19 CTs preliminarily assessed by the CTS in the timeframe considered in this analysis, officially registered with a unique EudraCT number and officially submitted, is 119. We analyzed the quality data and the quality-assessment outcome regarding the 119 CTs received at the CTO. The documentation submitted and the quality-assessment activity performed on these CTs represent the source of the data in scope for this research.

During the selected period, some CTs were resubmitted with the same EudraCT number upon a first negative outcome and were ultimately authorized. For a few others, the initial negative outcome was instead reconfirmed during a second submission. The overall number of requests for authorization of CTs is therefore greater than the relevant number of EudraCT codes; however, for the purpose of this research, the quality documentation and its assessment is considered only once for each EudraCT number.

Quality issues raised before a positive or negative decision, or before the withdrawal of a CT, were considered.

We retrieved, collected, and analyzed the quality documentation available in the chemistry manufacture and control (CMC) section of the IMPD, for any test IMP, as declared in the CTA form [20]. For some CTs, multiple IMPs were declared to be under test; therefore, the number of IMPs is higher than the number of EudraCTs officially received.

It is important to note that medicinal products with a marketing authorization may also be investigated in a CT. They are classified as IMPs as far as they are considered a test or reference substance because their packaging or formulation may differ from the authorized ones, because it is necessary to obtain additional safety information on the authorized form, or because they are intended to be tested for repurposing in a different therapeutic area or condition, such as in the case of COVID-19. In addition, medicinal products used as comparators or placebos are also considered IMPs. However, placebos and comparators are not included in the scope of this research, which is limited to IMPs declared as a test in section D of the CTA form, regardless of whether a marketing authorization is available for another indication. From the quality point of view, the substantial difference is that a consolidated quality and safety profile will already be available for the authorized products and therefore, in accordance with the Italian Minister of Health Decree dated 21 December 2007 and its updates [21], a full IMPD is not mandatory and may not be provided. A S-IMPD can instead be submitted to support the safety profile or even a SmPC as reference safety information for registered, non-modified products.

## 5. Conclusions

This is the first time that at the CTO in AIFA, quality documentation and data provided by sponsors in the CTAs are retrieved and analyzed, and that quality-assessment results are discussed, providing valuable and transparent information on the quality profile of IMPs. In addition, the focus is put on CTs with a COVID-19 indication submitted during a world health emergency that stressed sponsors’ and regulatory authorities’ procedures and challenged the regulatory framework.

With this research, we confirm that for those CTs that were assessed and authorized, chemistry manufacturing and control documentation, GMP compliance, data, and information provided to the CTO of AIFA as part of a COVID-19 CT application, from March 2020 to March 2021, are supporting and meet quality standards, regardless of the type of clinical trial, commercial or non-commercial. The assessment process in place based on requests for information and prompt responses from the sponsor, in conjunction with the commitment and experience of the assessors, withstood the impact of the state of emergency, continuing to ensure a good reactivity, and the results highlight the role of the assessor in the protection of public health. This research highlighted differences between commercial and non-commercial sponsors. Even if more non-commercial than commercial COVID-19 CTs were submitted in the first year of the pandemic, the majority of quality issues were raised in the context of the assessment of commercial studies. However, in terms of quality documentation, full IMPDs were mainly presented in commercial CTs, and SmPCs mainly in non-commercial ones. Non-commercial CTs usually test IMPs with a consolidated quality and safety profile, and this explains and confirms the expected lower frequency in quality issues detected.

Quality issues were, in general, noticed across almost all sections of the IMPD, denoting that quality standards and the level of compliance in the documentation submitted by all sponsors should definitely be increased. Stability data, specifications, GMP compliance, CTA form compliance, and control of materials areas were identified as critical for quality. Issues with batch analyses, labeling, impurities, quality documentation compliance, and description of the manufacturing process and process controls were also identified as sectors with a major impact on quality. In addition, non-commercial sponsors should particularly focus on CTA form compliance, GMP compliance, quality documentation compliance, and labeling. Other areas of attention were identified and must still be carefully monitored; however, the frequency of issues observed was lower.

Regardless of the health emergency period, the same legal requirements for safety, efficacy, and quality apply to COVID-19 IMPs, including vaccines, such as for any other medicinal product assessed during the evaluation of the benefit–risk balance of a CT application. It is important to underline that there is no evidence of any lowering in quality or safety standards during the evaluation of a COVID-19 CT application.

## Figures and Tables

**Table 1:** COVID-19 CTs assessed from March 2020 to March 2021 and their quality assessment outcome per study type.

COVID-19 CTs Assessed	Number of CTs (%) with (Yes)/without (No) Quality Issues		Study Type	Number of CTs per Quality Assessment Outcome and Study Type (%)
119	Yes	85 (71.43%)	Commercial	41 (34.45%)
			Non-commercial	44 (36.98%)
	No	34 (28.57%)	Commercial	10 (8.40%)
			Non-commercial	24 (20.17%)

## Data Availability

Additional information on the data presented in this study is available on request from the corresponding author. The data are not publicly available due to the protection of commercially confidential information.

## Appendix A

**Table A1:** Number of quality issues and their classification for COVID-19 CTs assessed from March 2020 to March 2021.

Classification Label of Quality Issues		Totals per Classification	Study Type	Totals per Classification and Study Type
CTA form compliance		49	Commercial	20
			Non-commercial	29
Quality documentation compliance (IMPD, S-IMPD, SmPC, CE mark)		27	Commercial	12
			Non-commercial	15
GMP compliance: information about all manufacturers involved (drug substance, drug product) and evidence of GMP (manufacturing licences/GMP certificates, QP declarations, CEPs provided)		62	Commercial	42
			Non-commercial	20
Drug Substance (DS)				
General information	Nomenclature	0	n/a	0
	Structure	5	Commercial	5
	General properties	11	Commercial	11
	Biological properties	2	Commercial	1
			Non-commercial	1
Manufacture	Description of manufacturing process and process controls	10	Commercial	10
	Control of materials	48	Commercial	44
			Non-commercial	4
	Control of critical steps and intermediates	11	Commercial	10
			Non-commercial	1
	Process validation and/or evaluation	1	Commercial	1
	Manufacturing process development	17	Commercial	16
			Non-commercial	1
Characterization	Elucidation of structure and other characteristics	13	Commercial	10
			Non-commercial	3
	Impurities	29	Commercial	26
			Non-commercial	3
Control of drug substance	Specifications	26	Commercial	26
	Analytical procedures	8	Commercial	8
	Validation of analytical procedures	12	Commercial	11
			Non-commercial	1
	Batch analyses	16	Commercial	16
	Justification of specification(s)	9	Commercial	9
Reference standards or materials		10	Commercial	10
			Non-commercial	1
Container closure system		4	Commercial	4
Stability		39	Commercial	34
			Non-commercial	5
Drug Product (DP)				
Description and composition of the investigational medicinal product		8	Commercial	7
			Non-commercial	1
Pharmaceutical development		7	Commercial	7
Manufacture	Batch formula	2	Commercial	2
	Description of manufacturing process and process controls	17	Commercial	14
			Non-commercial	3
	Controls of critical steps and intermediates	6	Commercial	5
			Non-commercial	1
	Process validation and/or evaluation	6	Commercial	4
			Non-commercial	2
Control of Excipients		7	Commercial	3
			Non-commercial	4
Control of Drug Product	Specifications	40	Commercial	38
			Non-commercial	2
	Analytical procedures	3	Commercial	2
			Non-commercial	1
	Validation of analytical procedures	12	Commercial	11
			Non-commercial	1
	Batch analyses	22	Commercial	19
			Non-commercial	3
	Characterization of impurities	0	n/a	0
	Justification of specification(s)	5	Commercial	4
			Non-commercial	1
Reference standards or materials		2	Commercial	2
Container closure system		11	Commercial	10
			Non-commercial	1
Stability		50	Commercial	40
			Non-commercial	10
Labeling		38	Commercial	12
			Non-commercial	26
Adventitious agents safety		19	Commercial	19
TOTAL		665	Commercial	525
			Non-commercial	140
